# Resistance towards and biotransformation of a *Pseudomonas*-produced secondary metabolite during community invasion

**DOI:** 10.1093/ismejo/wrae105

**Published:** 2024-06-14

**Authors:** Morten L Hansen, Zsófia Dénes, Scott A Jarmusch, Mario Wibowo, Carlos N Lozano-Andrade, Ákos T Kovács, Mikael L Strube, Aaron J C Andersen, Lars Jelsbak

**Affiliations:** Department of Biotechnology and Biomedicine, Technical University of Denmark, Søltofts Plads bldg. 221, DK-2800 Kgs Lyngby, Denmark; Department of Biotechnology and Biomedicine, Technical University of Denmark, Søltofts Plads bldg. 221, DK-2800 Kgs Lyngby, Denmark; Department of Biotechnology and Biomedicine, Technical University of Denmark, Søltofts Plads bldg. 221, DK-2800 Kgs Lyngby, Denmark; Department of Biotechnology and Biomedicine, Technical University of Denmark, Søltofts Plads bldg. 221, DK-2800 Kgs Lyngby, Denmark; Department of Biotechnology and Biomedicine, Technical University of Denmark, Søltofts Plads bldg. 221, DK-2800 Kgs Lyngby, Denmark; Department of Biotechnology and Biomedicine, Technical University of Denmark, Søltofts Plads bldg. 221, DK-2800 Kgs Lyngby, Denmark; Department of Biotechnology and Biomedicine, Technical University of Denmark, Søltofts Plads bldg. 221, DK-2800 Kgs Lyngby, Denmark; Department of Biotechnology and Biomedicine, Technical University of Denmark, Søltofts Plads bldg. 221, DK-2800 Kgs Lyngby, Denmark; Department of Biotechnology and Biomedicine, Technical University of Denmark, Søltofts Plads bldg. 221, DK-2800 Kgs Lyngby, Denmark

**Keywords:** DAPG, pyoluteorin, orfamide A, SynCom, microbial interactions, Pseudomonas invasion, biotransformation, secondary metabolites

## Abstract

The role of antagonistic secondary metabolites produced by *Pseudomonas protegens* in suppression of soil-borne phytopathogens has been clearly documented. However, their contribution to the ability of *P. protegens* to establish in soil and rhizosphere microbiomes remains less clear. Here, we use a four-species synthetic community (SynCom) in which individual members are sensitive towards key *P. protegens* antimicrobial metabolites (DAPG, pyoluteorin, and orfamide A) to determine how antibiotic production contributes to *P. protegens* community invasion and to identify community traits that counteract the antimicrobial effects. We show that *P. protegens* readily invades and alters the SynCom composition over time, and that *P. protegens* establishment requires production of DAPG and pyoluteorin. An orfamide A-deficient mutant of *P. protegens* invades the community as efficiently as wildtype, and both cause similar perturbations to community composition. Here, we identify the microbial interactions underlying the absence of an orfamide A mediated impact on the otherwise antibiotic-sensitive SynCom member, and show that the cyclic lipopeptide is inactivated and degraded by the combined action of *Rhodococcus globerulus* D757 and *Stenotrophomonas indicatrix* D763. Altogether, the demonstration that the synthetic community constrains *P. protegens* invasion by detoxifying its antibiotics may provide a mechanistic explanation to inconsistencies in biocontrol effectiveness in situ.

## Introduction

The use of bacteria and their antimicrobial metabolites has emerged as a potential alternative to maurhofer 1994synthetic agrochemicals in control of phytopathogens. In *Pseudomonas protegens*, a model organism for biocontrol, secondary metabolites such as 2,4-diacetylphloroglucinol (DAPG), pyoluteorin, and orfamide A have been shown to play essential roles in suppression of plant pathogens [1–5]. For example, DAPG suppress the fungal take-all disease in cereal plants [6], pyoluteorin is involved in the suppression of *Pythium* damping-off of cress [4], and orfamide A can reduce bacterial wilt disease in tomato [7]. However, as bacterial biocontrol is often associated with varying efficiencies across fields [8] and plants [4], it remains an important challenge to identify and mitigate the processes responsible for these variations.

Although biocontrol is a complex phenomenon that relies on multiple processes for its effectiveness [9], central processes involve interactions between an invading biocontrol strain and its secreted antibiotics, and the resident microbial community. For example, invasion and competition with existing microbial populations in the soil and rhizosphere is a prerequisite for effective biocontrol. Although recent results have shown that specific *Pseudomonas*-produced secondary metabolites are indeed required for efficient invasion of the rhizosphere of *Arabidopsis* roots and alters the structure of the resident, synthetic microbial community (SynCom) [10], other studies have found microbial communities that are more resilient towards invasion. For example, earlier studies have shown that *P. protegens* inoculants, including engineered variants with enhanced production of DAPG and pyoluteorin, had no impact on the structure of the bacterial community on cucumber roots or on the proportion of bacteria that were tolerant or sensitive to DAPG and pyoluteorin [11]. Similarly, inoculation with various species of secondary metabolite producing fluorescent *Pseudomonas* may result in vastly different outcomes, ranging from temporary, spatially limited, and transient effects on the natural rhizosphere microbiomes [12–15] to significant perturbations within indigenous microbiomes [16, 17]. Although these results suggest that resilience towards invading *Pseudomonas* biocontrol strains and their antibiotics may depend on the composition and activity of the invaded microbial community, little is known about the underlying community traits or mechanisms that may operate to tolerate otherwise toxic levels of antibiotics and to constrain *Pseudomonas* invasion.

In this study we use a hydrogel-based bead system [18] as in vitro model system to systematically explore the contribution of the secondary metabolites, DAPG, pyoluteorin, and orfamide A, to the ability of *P. protegens* DTU9.1 to invade and establish in a four-membered SynCom of commonly isolated soil bacteria [19, 20]. The hydrogel environment has been shown to mimic soil characteristics allowing for spatial distribution of microbes, as well as surface colonization [18], and thus enabling systematic analyses of community-level interactions affecting secondary metabolism in *P. protegens* DTU9.1 in an artificial yet soil-like environment. We showed that community traits affect SynCom susceptibility towards the toxic antibiotics, and that one of the underlying mechanisms is hydrolysis and subsequent degradation of the cyclic lipopeptide orfamide A involving several members of the four-species SynCom. These results provide insight into how levels and activity of antibiotic metabolites from invading *Pseudomonas* strains may be shaped by interspecies interactions in microbial communities and provide a framework for studying community mechanisms that affect invasion or efficacy of biocontrol strains.

## Methods

### Microorganisms and cultivation

Plasmid cloning was performed in *Escherichia coli* CC118-*λpir*. Cells were cultured in lysogeny broth (LB; Lennox, Merck, St. Louis, MO, USA) with appropriate antibiotics. The antibiotic concentration used was 10 μg/mL for chloramphenicol, and 8 μg/mL and 50 μg/mL for tetracycline for *E. coli* and *P. protegens*, respectively. *E. coli* CC118 λ*pir* was cultured by inoculating a single colony in 5 mL LB broth and incubating overnight at 37°C with shaking (200 rpm). *P. protegens* DTU9.1 and members of the synthetic community were cultured by inoculating a single colony in 5 mL LB broth and incubating overnight at 30°C with shaking (200 rpm). The community members include *Pedobacter* sp*.* D749 (Accession: CP079218), *Rhodococcus globerulus* D757 (Accession: CP079698), *S. indicatrix* D763 (Accession: CP079106), and *Chryseobacterium* sp*.* D764 (Accession: CP079219) [19].

### Minimal inhibitory concentration (MIC) assay

A MIC assay was conducted to determine the susceptibility of each SynCom member towards orfamide A. SynCom members were cultured in four biological replicates in LB O/N. Cells were washed twice in 0.9% NaCl. A clear 96-well flat-bottom microplate (Greiner Bio-One) was prepared with 200 μL 0.1x TSB per well inoculated with bacteria to an initial OD_600_ of 0.01 and appropriate serial dilutions of metabolites. The microplate was covered with semi-permeable membrane (Breathe-Easy, Merck) and incubated at room temperature with 600 rpm shaking for 24 hours, followed by MIC-value determination.

### Generation of secondary metabolite deficient mutants

To generate mutants in *P. protegens* DTU9.1 incapable of synthesizing DAPG, pyoluteorin, and orfamide A, genes required for biosynthesis were deleted by allelic replacement as reported previously [21]. Primers used for cloning and verification are summarized in Supplementary Table 1. In short, DNA fragments directly upstream and directly downstream of *the gene of interest* were PCR amplified and subsequently joined by splicing-by-overlap extension PCR with XbaI and SacI overhangs. The purified PCR product was restriction-digested and inserted in pNJ1 [22]. The resulting plasmid was mobilized into *P. protegens* DTU9.1 via triparental mating with *E. coli* HB101 harboring the helper plasmid pRK600. Merodiploid transconjugants were initially selected on *Pseudomonas* Isolation Agar (PIA, Merck) supplemented with 50 μg/mL tetracycline. A second selection was performed on NSLB agar (10 g/L tryptone, 5 g/L yeast extract, 15 g/L Bacto agar) with 15% v/v sucrose. Candidates for successful deletion were confirmed by PCR and verified by Sanger sequencing at Eurofins Genomics.

### Integration of *P. protegens* DTU9.1 in a synthetic microbial community

The effect of introducing *P. protegens* DTU9.1 into a synthetic bacterial community was investigated in an artificial soil medium composed of spherical hydrogel beads. The beads were prepared according to a previously published method [18]. In short, a polymer solution was prepared as a 4:1 mixture of 9.6 g/L gellan gum (Phytagel, Sigma) and 2.4 g/L sodium alginate (Sigma) dissolved in distilled water. Spherical beads with a diameter of approximately 3–4 mm were formed by dropping polymer solution into a cross-linker solution containing 20 g/L CaCl_2_ with a 10 mL syringe. Then, the beads were soaked in 0.1x TSB (Sigma) for 1 hour followed by sieving the beads to remove residual TSB medium. Finally, 20 mL beads were transferred to 50 mL Falcon tubes. Cultures of the four community members and *P. protegens* WT and Δ*ofaA* were grown overnight (O/N). The optical density at 600 nm (OD_600_) of *Pedobacter* and *Rhodococcus* was set to 2.0, for *Stenotrophomonas* and *Chryseobacterium* it was set to 0.1, and for *P. protegens* DTU9.1 and mutants it was set to 0.001 (see Fig. 2A for CFU/mL). Bacterial inoculation suspensions were prepared by mixing equal volumes in a total volume of 2 mL 0.1x TSB. Lastly, the prepared beads were inoculated with the 2 mL bacterial suspension. Inoculated bead systems were incubated static at RT and samples collected after 1, 4, and 7 days. Sampling was performed by briefly shaking the bead systems followed by extracting approximately 1 mL beads into new 15 mL Falcon tubes. Extracted beads were subsequently diluted in 0.9% (w/v) NaCl according to their weight to normalize the amount of bacterial cells. The tubes were shaken on a vortex for 10 minutes at maximum speed to disrupt the hydrogel beads. After vortexing, dilutions were spread on 0.1x TSA plates and incubated at RT for 48 hours before counting CFU/mL. The remaining liquid (approx. 5 mL) of the processed samples were saved for chemical detection of secondary metabolites.

### Detection of secondary metabolites with LC-HRMS

To extract secondary metabolites in the hydrogel bead samples and supernatants of O/N cultures, an equal volume of ethyl acetate was added to the samples followed by shaking the tubes briefly. For extraction of metabolites from 0.1x TSA plates, an agar plug covering entire bacterial colonies (approx. 6 mm diameter for normal plates and 30 mm diameter for swarming plates) was suspended in 1 mL isopropanol:ethyl acetate (1:3 v/v) with 1% formic acid and shaken briefly. For both types of extractions, tubes were subsequently centrifuged for 3 minutes at 5000 x g and the top layer was transferred to new tubes. Extracts were then evaporated under N_2_. The dried extracts were re-suspended in 200 μL methanol (MeOH) and centrifuged for 3 minutes at 13000 x g. The supernatant was transferred to HPLC vials and subjected to ultra high-performance liquid chromatography electrospray ionization time-of-flight mass spectrometry (UHPLC-HRMS) analysis.

LC-HRMS was performed on an Agilent Infinity 1290 UHPLC system. Liquid chromatography of 1 μL or 5 μL extract was performed using an Agilent Poroshell 120 phenyl-C_6_column (2.1 × 150 mm, 1.9 μm) at 60°C using CH_3_CN and H_2_O, both containing 20 mM formic acid. Initially, a linear gradient of 10% CH_3_CN/H_2_O to 100% CH_3_CN over 10 min was employed, followed by isocratic elution of 100% CH_3_CN for 2 min. Then, the gradient was returned to 10% CH_3_CN/H_2_O in 0.1 min and finally isocratic condition of 10% CH_3_CN/H_2_O for 1.9 min, all at a flow rate of 0.35 min/mL. HRMS data was recorded in positive ionization on an Agilent 6545 QTOF MS equipped with an Agilent Dual Jet Stream electrospray ion (ESI) source with a drying gas temperature of 250°C, drying gas flow of 8 min/L, sheath gas temperature of 300°C and sheath gas flow of 12 min/L. Capillary voltage was 4000 V and nozzle voltage was set to 500 V. Fragmentation data was collected using auto MS/MS at three collision energies (10, 20, 40 eV). The HRMS data was processed and analyzed using Agilent MassHunter Qualitative Analysis B.07.00. HPLC grade solvents (VWR Chemicals) were used for extractions whereas LCMS grade solvents (VWR Chemicals) were used for LCMS.

### GNPS molecular networking

A molecular network was created using the Feature-Based Molecular Networking [23] workflow on GNPS (https://gnps.ucsd.edu, [24]). The workflow run can be found at this link: https://gnps.ucsd.edu/ProteoSAFe/status.jsp?task=1ad207802221433ca5431d22f2638d0e. Raw data was processed using MZmine2.53 [25]. Data was filtered by removing all MS/MS fragment ions within +/− 17 Da of the precursor m/z. MS/MS spectra were window filtered by choosing only the top 6 fragment ions in the +/− 50 Da window throughout the spectrum. Additional settings include: precursor ion mass tolerance was set to 0.05 Da, MS/MS fragment ion tolerance to 0.05 Da, and edges were filtered to have a cosine score above 0.7 and more than 10 matched peaks. The spectra in the network were then searched against GNPS spectral libraries [24, 26]. The library spectra were filtered in the same manner as the input data. All matches kept between network spectra and library spectra were required to have a score above 0.7 and at least 6 matched peaks. The DEREPLICATOR was used to annotate MS/MS spectra [27]. The molecular networks were visualized using Cytoscape 3.8.2 [28].

### MALDI sample preparation

Bacterial strains were cultured on 10 mL 0.1x TSA plates at 22°C. After 4 days of incubation, the microbial colony and surrounding agar was sectioned and mounted on an IntelliSlides conductive tin oxide glass slide (Bruker). The sample was covered with matrix by spraying 1.75 mL of a matrix solution in a nitrogen atmosphere. The matrix solution was 2,5-dihydrobenzoic acid (DHB) of 20 mg/mL concentration in ACN/MeOH/H_2_O (70:25:5, v/v/v) according to [29].

### MALDI mass spectrometry imaging (MSI)

Samples were dried in a desiccator overnight prior to MSI measurement. The samples were then subjected to timsTOF flex mass spectrometer (Bruker) for MALDI-IMS acquisition. Calibration was done using red phosphorus. The samples were run in positive MS scan mode with 100 μm raster width and a *m/z* range of 100–2000. Briefly, a photograph of the colonies was loaded onto Fleximaging software, three teach points were selected to align the background image with the sample slide, measurement regions were defined, and the automatic run mode was then employed. The settings in the timsControl were as follow: Laser: imaging 100 μm, Power Boost 3.0%, scan range 26 μm in the XY interval, and laser power 90%; Tune: Funnel 1 RF 300 Vpp, Funnel 2 RF 300 Vpp, Multipole RF 300 Vpp, isCID 0 eV, Deflection Delta 70 V, MALDI plate offset 100 V, quadrupole ion energy 5 eV, quadrupole loss mass 100 *m/z*, collision energy 10 eV, focus pre TOF transfer time 75 μs, pre-pulse storage 8 μs. After data acquisition, the data was analyzed using SCiLS software.

### Swarming assay

For the swarming assay *R. globerulus* D757, as well as the two variants of *P. protegens* DTU9.1 (WT and Δ*ofaA*) were cultured in three biological replicates in LB broth O/N. Cells were washed twice in 0.9% NaCl prior to adjusting OD_600_ to 1 for *Rhodococcus* and 0.001 for *Pseudomonas*. For cocultures equal volumes of culture suspensions were mixed, whereas for axenic plates cell suspensions were mixed with an equal volume of 0.9% NaCl. Aliquots of 5 μL were spotted in the center of 0.1x TSA plates with 0.6% agar. Plates were incubated for 48 hours at 30°C prior to taking pictures. Swarming areas were analyzed with ImageJ.

### Chemical hydrolysis of orfamide A

Pure orfamide A (Cayman, United States) was chemically linearized by hydrolysis by mixing 100 μL (0.386 μmol, suspended in methanol) and 193 μL 0.1 M aqueous LiOH (1.93 μmol, 5 equimolar). The solution was stirred at room temperature for 21 h. The reaction mixture was quenched by addition of 29.3 μL 1 M HCl. This led to the formation of a white precipitate, which was re-dissolved by addition of 677.7 μL methanol. Complete hydrolysis was verified by LC-HRMS.

### Statistics

Multivariate analysis of community composition was performed using PERMANOVA on Bray-Curtis dissimilarities and the model formulation Y ~ Time + Variant + Time:Variant. Follow-up PERMANOVAs were performed on each time point with only Variant as the dependent variable. A univariate comparison of CFU counts and metabolite concentrations in SynCom versus axenic culture of *P. protegens* DTU9.1 was carried out using Student’s *t*-tests assuming equal variance.

### Data availability

LC-HRMS data has been deposited at MassIVE with the identifier, MSV000092145. MALDI-MSI has been uploaded to Metaspace (https://metaspace2020.eu/project/Hansen-2023). Demultiplexed 16S rRNA sequencing reads were uploaded to NCBI SRA database under BioProject number PRJNA983551.

## Results

### Utilizing a hydrogel bead system to explore invasion of *P. protegens* DTU9.1 in a synthetic microbial community

To explore the microbial interactions and their effects on community composition over time during invasion of *P. protegens* DTU9.1, a porous hydrogel bead system was chosen as cultivation system, as it has been shown to mimic soil characteristics and allow for spatial distribution of microbes [18, 30]. The four-membered SynCom (*Pedobacter* sp. D749, *R. globerulus* D757, *Stenotrophomonas indicatrix* D763, and *Chryseobacterium* sp. D764) was comprised of co-isolated species from a soil sample site that we previously demonstrated to also contain *P. protegens* [31].

**Figure 1:**
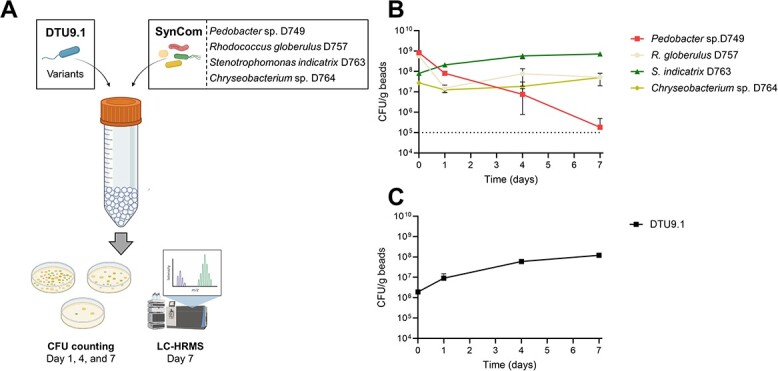
**Utilizing hydrogel beads as in vitro model system to explore *pseudomonas*-invasion in a synthetic microbial community*.* A)** Experimental setup of *Pseudomonas*-invasion in the hydrogel bead system with a four-membered synthetic microbial community (SynCom). Community composition was determined by sampling after 1, 4, and 7 days. Metabolite profiles were determined with LC-HRMS after terminating the experiment on day 7. **B)** the stability of the SynCom alone in the hydrogel bead system was evaluated. The dotted line represents the limit of detection of colony forming units at 10^5^ CFU/mL. **C)** Wildtype *P. protegens* DTU9.1 proliferates in the hydrogel beads when cultivated axenically. Data in (**B**) and (**C**) was derived from three biological replicates.

Initially, it was verified that the four SynCom members could establish and co-exist in the hydrogel beads over a 7-day period (Fig. 1B). As shown, all members could be detected, although *Pedobacter* sp*.* D749 reached an abundance close to the limit of detection (10^5^ CFU/mL). Additionally, it was determined that *P. protegens* DTU9.1 could colonize and maintain itself in the hydrogel bead environment when cultivated axenically (Fig. 1C). After four days of cultivation, *Pseudomonas* reached a plateau of approximately 10^8^ CFU/mL, which was maintained at the seventh and final day of the experiment. Lastly, it was confirmed that wildtype *P. protegens* DTU9.1 produced detectable amounts of DAPG, pyoluteorin, and orfamide A after seven days of axenic cultivation in the bead system, as verified using liquid chromatography coupled to high resolution-mass spectrometry (LC-HRMS) (Fig. S1).

### *P. protegens* DTU9.1 invades a four-membered synthetic microbial community in a soil-mimicking environment

Next, we sought to investigate the sensitivity of each SynCom member towards the three antimicrobial *Pseudomonas*-produced metabolites (DAPG, pyoluteorin, and orfamide A). *R. globerulus* D757 displayed sensitivity towards all three metabolites, whereas three members (*Pedobacter* sp. D749, *R. globerulus* D757 and *Chryseobacterium* sp. D764) were equally susceptible to pyoluteorin with an MIC of 8 μg/mL (Table 1). *Stenotrophomonas indicatrix* D763 was generally more resistant towards all three antimicrobial metabolites (Table 1). Thus, we hypothesized that several community members would be affected upon invasion by wildtype *P. protegens* DTU9.1 given its ability to produce all three metabolites in the hydrogel bead system.

**Table 1:** Minimal inhibitory concentrations of DAPG, pyoluteorin, and orfamide A against the SynCom members.

	**Minimal Inhibitory Concentration** **(μg/ml)**		
	**DAPG**	**Pyoluteorin**	**Orfamide A**
*Pedobacter* sp. D749	16	8	> 64
*Rhodococcus globerulus* D757	8	8	16
*Stenotrophomonas indicatrix* D763	> 64	16	> 64
*Chryseobacterium* sp. D764	64	8	> 64

**Figure 2:**
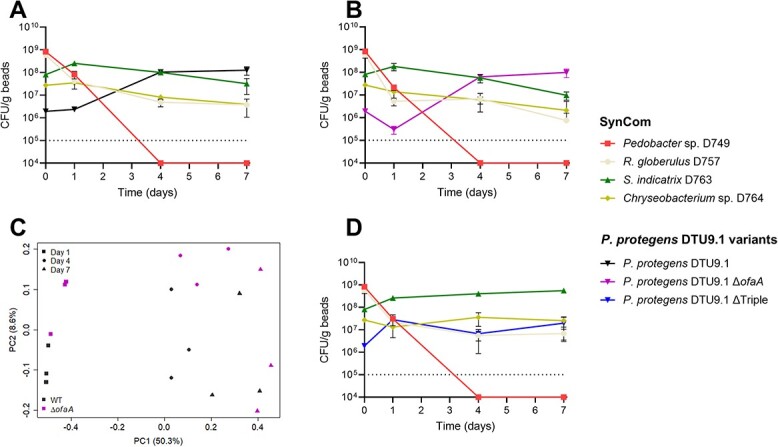
***P. protegens* DTU9.1 invades a four-membered synthetic microbial community in a soil-like environment*.*** Abundance was determined as colony forming units in the hydrogel bead system of each SynCom member and the introduced *P. protegens* DTU9.1 variant; WT (**A**), Δ*ofaA* (**B**), and ΔTriple (**D**) after 1, 4, and 7 days. The dotted line represents the limit of detection of colony forming units at 10^5^ CFU/mL. Data was derived from three biological replicates. **C)** A principal coordinate analysis (PCoA) was performed on CFU data including all bacterial species of the systems inoculated with *P. protegens* DTU9.1 WT and Δ*ofaA* to compare the effects of sampling time (symbols) and the variants of *P. protegens* DTU9.1 (color) on the bacterial composition in a soil-like environment.

To test this hypothesis, we cultured *P. protegens* DTU9.1 wildtype along with the four-membered SynCom for seven days in the hydrogel bead system. After one day of cultivation, all SynCom members were still detected (Fig. 2A), whereas the amount of cultivable *P. protegens* DTU9.1 cells were 100–1000 fold lower than the SynCom members, likely owing to the low inoculum size of *P. protegens* DTU9.1 (see Methods). However, on the fourth day, *P. protegens* DTU9.1 had reached high cell numbers (≈10^8^ CFU/mL), which were maintained throughout the experiment and comparable to that observed in axenic cultivation (c.f. Figure 1C). On the seventh day of incubation *P. protegens* DTU9.1 constituted the majority of the bacterial biomass, although three of the four SynCom members remained detectable with our method of colony counting (Fig. 2A).

On the fourth day and onwards, colony forming units of *Pedobacter* sp. D749 were no longer detectable in the systems inoculated with *P. protegens* DTU9.1. However *Pedobacter* sp. D749 could be detected at higher cell titers throughout the experiment without the addition of *Pseudomonas*, albeit with cell counts close to the detection limit on the seventh day (Fig. 1B). We collected DNA from the hydrogel bead systems for each sampling point and performed amplicon sequencing of the 16S rRNA gene to verify the presence of *Pedobacter* sp*.* D749. The presence of the bacterium was confirmed throughout the seven days in both the control system and the system with *P. protegens* DTU9.1 wildtype (Fig. S2).

To investigate further if the changes in community composition upon invasion by *P. protegens* DTU9.1 was due to production of toxic levels of the three antibiotics, we extracted metabolites from the hydrogel bead systems after seven days of incubation and analyzed the samples with LC-HRMS to verify the production and quantity of the three secondary metabolites in question. During axenic cultivation, *P. protegens* DTU9.1 reached similar levels of colony forming units as compared to growth alongside the SynCom (Table 2).

**Table 2:** Concentration of secondary metabolites from *P. protegens* DTU9.1 after 7 days of growth in a soil-mimicking environment.

	Log_10_ CFU/ml^a^ *Pseudomonas*	Secondary metabolite (μg/ml) Mean ± Standard deviation		
		**DAPG**	**Pyoluteorin**	**Orfamide A**
*P. protegens* DTU9.1	8.07 ± 0.11	3.96 ± 0.82	5.85 ± 0.56	25.58 ± 2.11
*P. protegens* DTU9.1 + SynCom	8.08 ± 0.15	9.16 ± 1.30	10.12 ± 2.19	3.87 ± 0.75
Statistical significance^b^	n.s.	*P* = 0.0087	*P* = 0.0556	*P* = 0.0002

a CFU/mL of *P. protegens* DTU9.1 either grown alone or with the SynCom for seven days in the bead system.

b Significance of the difference in secondary metabolite concentration was analyzed by Student’s *t*-test.

The concentrations of the secondary metabolites produced by *P. protegens* DTU9.1 differed markedly between the two systems. In the case of both DAPG and pyoluteorin, concentrations were elevated during cocultivation compared to axenic growth, although this was not significant for pyoluteorin (*P* = 0.056). This could suggest that *P. protegens* DTU9.1 responded to the presence of competing microorganisms by increasing the production of its antimicrobial secondary metabolites (i.e. DAPG and pyoluteorin). However, in the case of the cyclic lipopeptide, orfamide A, the concentration was significantly lower (*P* = 0.0002) when *P. protegens* DTU9.1 was cultivated with the SynCom compared to axenic growth (Table 2). Considering that the level of orfamide A in the SynCom system is below the measured MIC values (Table 1), we hypothesized that introducing an orfamide A-deficient mutant would not cause significant perturbations to the system compared to introducing wildtype *P. protegens* DTU9.1. Thus, to test this, we constructed an orfamide A-deficient mutant (Δ*ofaA*) and introduced it into the SynCom (Fig. 2B). Expectedly, the changes to community composition over a 7-day period were similar to the system inoculated with wildtype *P. protegens* DTU9.1. The overall effect on community composition over time compared between the two systems (SynCom + WT and SynCom + Δ*ofaA*) was evaluated with a principal coordinate analysis (PCoA) using Bray-Curtis dissimilarities (Fig. 2C). The analysis suggested that the major factor affecting abundance was the sampling time, as separate clusters representing the three sampling times appeared across the first principal component, which explained the largest portion of the variance in the data (50.3%). An overall PERMANOVA using sampling time, genotypic variant of invading *P. protegens* DTU9.1, and their interaction as fixed effects further confirmed the significance of sampling time on the community composition (*P* = 9.99 ∙ 10^−5^, *R*^2^ = 0.75). A growth assay of each SynCom member, as well as *P. protegens* DTU9.1 WT and Δ*ofaA* in liquid broth revealed that the invading *Pseudomonas* had a significantly faster doubling time than all SynCom members (Fig. S3), whereas *Pedobacter* sp. D749 had the longest doubling time. This could explain the observed significant effect of sampling time on community composition, as visualized by the PCoA (Fig. 2C).

The levels of both DAPG and pyoluteorin were elevated during coculture of *P. protegens* DTU9.1 and the SynCom (Table 2). Furthermore, these levels exceeded the MIC values towards several SynCom members (Table 1). Thus, to investigate if all three antimicrobial metabolites played a role in the ability of *P. protegens* DTU9.1 to establish within the bead system co-inoculated with the SynCom, we constructed a Δ*phlACB*, Δ*pltA*, Δ*ofaA* (from here on referred to as ΔTriple) knockout mutant deficient in production of DAPG, pyoluteorin, and orfamide A. The ΔTriple mutant was not able to establish and reach cell titers comparable to that of *P. protegens* DTU9.1 wildtype (Fig. 2D). Furthermore, a PCoA and subsequent overall PERMANOVA confirmed that variant rather than sampling time was the main driver of perturbations to community composition over time when comparing the SynCom + WT and SynCom + ΔTriple systems (Fig. S4). However, introduction of mutants deficient in production of either DAPG or pyoluteorin (Δ*phlACB* and Δ*pltA*, respectively) into the SynCom caused similar perturbations to community composition over time as wildtype (Fig. S5A). Additionally, we observed that levels of DAPG and pyoluteorin increased in their respective opposite knockout mutant during axenic growth of *P. protegens* DTU9.1 (Fig. S5B). This could suggest that *P. protegens* DTU9.1 utilizes a secondary metabolite-mediated approach to establish within the SynCom, which relies on production of at least DAPG or pyoluteorin.

### Orfamide A is degraded during cocultivation of *P. protegens* DTU9.1 and SynCom in the hydrogel bead system

**Figure 3:**
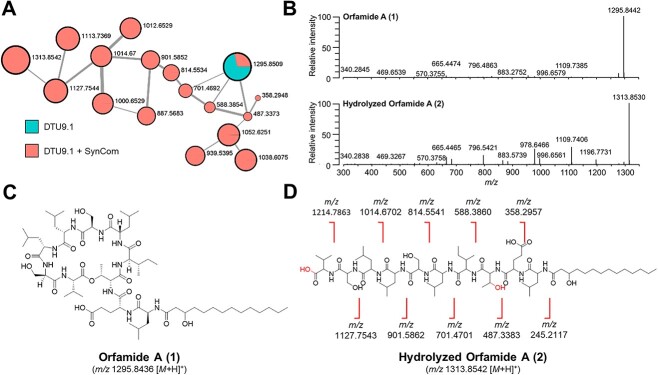
**Orfamide A is degraded during coculture of *P. protegens* DTU9.1 and SynCom in a soil-mimicking environment*.* A)** Molecular family of metabolites related to orfamide A derived from the molecular network (GNPS). Values next to nodes represent *m/*z-values and node size is scaled to metabolite mass. Colors inside nodes represent relative abundance in the system with axenically grown *P. protegens* DTU9.1 or the coculture between *P. protegens* DTU9.1 and the SynCom. Edges between nodes represent molecular similarity based on cosine scores. **B)** Observed fragmentation patterns of **1** and **2** revealed by tandem mass spectrometry (MS/MS). **C)** Structure of orfamide A (**1**). **D)** Structure of hydrolyzed orfamide A (**2**) and the calculated masses of each degradation product. Hydroxy-groups marked in red represent the outcome of hydrolyzing the ester bond of **1**.

We observed that the levels of orfamide A were significantly reduced during coculture of *P. protegens* DTU9.1 and the SynCom compared to axenic cultivation of *Pseudomonas* (Table 2). Given that one of the SynCom members is sensitive towards orfamide A and that orfamide A is required for swarming behavior to colonize new niches [32], our observation could indicate that members of the synthetic community either inhibited production of orfamide A or affected the persistence of the metabolite over time as a potential mechanism of resistance. To further investigate this observation and the fate of orfamide A (**1**, Fig. 3C) further, the MS data from the LC-HRMS analyses was subjected to Global Natural Product Social (GNPS) molecular network analysis [24] to identify potential chemical relationships between features across the three systems (Fig. S6). This revealed a distinct molecular family displaying the presence of orfamide A (*m/z* 1295.8509) during both axenic cultivation of *P. protegens* DTU9.1 and cocultivation with the SynCom (Fig. 3A). This molecular family also contained features with related fragmentation patterns to orfamide A that only appeared during cocultivation of *P. protegens* DTU9.1 and the SynCom. One feature, *m/z* 1313.8542, corresponded to the mass of hydrolyzed orfamide A, with the addition of 18 Da (Fig. 3A). Fragmentation analysis suggested hydrolysis of the ester bond connecting the macrocyclic ring (Fig. 3B), which created a linearized congener of orfamide A (**2**, Fig. 3D) when *P. protegens* DTU9.1 was cultivated alongside the SynCom in the bead system.

Potential degradation products of orfamide A were also present in the same molecular family (Fig. 3A). The suspected degradation products were verified in the LC-HRMS data of the extracts. Differences in the retention times of each of these features provided confirmation that they were not arising from in-source fragmentation (Fig. S7). Fragmentation patterns were subsequently analyzed, confirming degradation products emanating from the hydrolyzed ester bond (Fig. S8). A similar phenomenon was observed for orfamide B through the presence of degradation products (*m/z* 1113.7369, *m/z* 1000.6529, and *m/z* 887.5683 in Fig. 3A). The remaining degradation products of orfamide B were present in concentrations too low to be selected for MS/MS, but were observed in the raw data (available from the raw data. See Methods). Taken together, these results demonstrate that one or multiple SynCom members were able to hydrolyze orfamide A (and orfamide B) from *P. protegens* DTU9.1 and degrade the linearized lipopeptide.

### Linearization of orfamide A is caused by *R. globerulus* D757 and inhibits the motility of *P. protegens* DTU9.1

**Figure 4:**
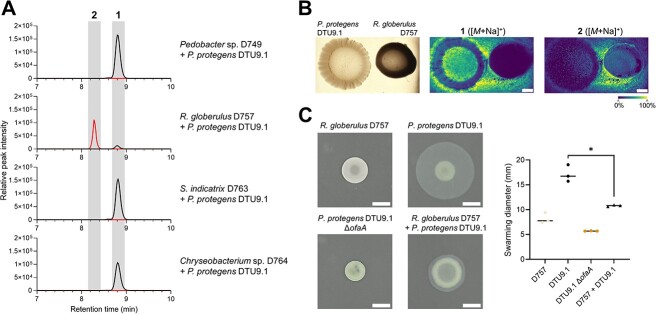
**Orfamide A is hydrolyzed by *R. globerulus* D757, which inhibits motility of *P. protegens* DTU9.1*.* A)** Extracted ion chromatograms (EIC) for **1** (*m/z* 1295.8436 ± 5 ppm) and **2** (*m/z* 1313.8542 ± 5 ppm) detected in agar plugs from co-cultures of *P. protegens* DTU9.1 and the respective SynCom member on 0.1x TSA plates. **B)** MALDI imaging of colonies of *P. protegens* DTU9.1 and *R. globerulus* D757 showing hydrolysis of orfamide A after 4 days of incubation. White scale bar represents 2 mm. **C)** Swarming assay on 0.1x TSA plates with 0.6% agar. *Left* displays representative images of three biological replicates. White scale bar represents 5 mm. *Right* shows the result of the three biological replicates. Statistical significance was determined with a Student’s *t*-test (*, *P* < 0.05).

To explore the degradation of orfamide A, we turned to dual-species interactions on agar surfaces to investigate if a single community member was responsible for linearization by hydrolysis and degradation. First, *P. protegens* DTU9.1 was cocultivated with each of the four SynCom members individually in mixed species colonies on 0.1x TSA. MS data of the metabolites extracted from an agar plug covering the entire bacterial colony revealed that the Gram-positive *R. globerulus* D757 was able to hydrolyze the ester bond of **1** yielding the linearized product, **2** (Fig. 4A). Additionally, we monitored the interaction between *P. protegens* DTU9.1 and *R. globerulus* D757 by matrix-assisted laser desorption-ionization mass spectrometry imaging (MALDI-MSI) to validate that the linearization of orfamide A indeed occurs in the interface between the two bacteria. The two species were cultivated for 4 days on 0.1x TSA plate prior to matrix application and imaging. Orfamide A (**1**) was secreted evenly around the *P. protegens* DTU9.1 colony, whereas the linearized product (**2**) was observed only in the interface between the two bacteria (Fig. 4B).

Additionally, we investigated the effect of coculturing *R. globerulus* D757 and *P. protegens* DTU9.1 on the swarming motility of the *Pseudomonas*. Axenic cultivation of *P. protegens* DTU9.1 led to a uniform faint circular spread from the point of inoculation after 48 hours of incubation (Fig. 4C). Expectedly, the Δ*ofaA* knockout mutant was completely impaired in the ability to swarm. Cocultivation with *R. globerulus* D757 significantly inhibited the swarming motility of wildtype *P. protegens* DTU9.1, where only a slight faint zone was observed surrounding the primary colony (Fig. 4C, see also Fig. S9). The presence of orfamide A (**1**) and its hydrolyzed congener (**2**) was verified by extracting metabolites from an agar plug covering the entire swarming area followed by LC-HRMS (Fig. S10A). Additionally, both variants of *P. protegens* DTU9.1 (WT and Δ*ofaA*) were chromosomally tagged with *gfp* under strong constitutive expression, which allowed us to determine the abundance of the non-fluorescent *R. globerulus* D757 and green fluorescent *P. protegens* DTU9.1 in the swarming colony by flow cytometry (Fig. S10B, Supplementary methods). This analysis showed that *P. protegens* DTU9.1 constituted half of the microbial biomass in the coculture with *R. globerulus* D757 after 48 hours, although swarming was inhibited. This suggests that hydrolysis of orfamide A by *R. globerulus* D757 may serve as both a defensive resistance mechanism to reduce toxic levels of the antimicrobial metabolite, while simultaneously blocking the swarming-mediated motility of the metabolite-producer, *P. protegens* DTU9.1.

### Fate of orfamide A is affected by a multi-species interaction involving *R. globerulus* D757 and *S. indicatrix* D763

**Figure 5:**
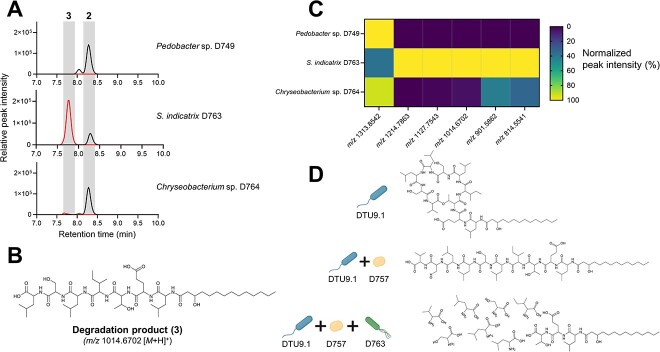
| **hydrolyzed orfamide A is further degraded by *S. indicatrix* D763*.* A)** Extracted ion chromatograms (EIC) for **2** (*m/z* 1313.8542 ± 5 ppm) and **3** (*m/z* 1014.6702 ± 5 ppm) detected in cultures supplemented with pre-hydrolyzed orfamide A after 24 hours of cultivation in liquid 0.1x TSB. **B)** Structure of the most abundant degradation product (**3**) with *m/z* value of 1014.6702. **C)** Normalized peak intensities of each degradation product detected in cultures from (**B**). Each value represents the mean peak intensity of two biological replicates. **D)** Model describing the fate of orfamide A in the presence of SynCom members.

In the dual species assays conducted above we only observed linearization of orfamide A by hydrolysis, but no subsequent degradation. Thus, we hypothesized that hydrolysis was a prerequisite for one or more of the remaining three SynCom members (*Pedobacter* sp. D749, *S. indicatrix* D763, and *Chryseobacterium* sp. D764) to further degrade the hydrolyzed orfamide A (**2**). To investigate this hypothesis, we chemically hydrolyzed pure orfamide A by prolonged incubation in an alkaline solution (see Methods) and exposed the remaining three SynCom members individually to hydrolyzed orfamide A over 24 hours in liquid broth. Subsequent extraction of metabolites from the supernatants revealed that *S. indicatrix* D763 could degrade the hydrolyzed lipopeptide (Fig. 5A). Here, we show the feature, *m/z* 1014.6702 (**3**), as an example of degradation. This feature corresponds to the mass of **2** excluding three amino acids from the C-terminal end (Fig. 5B). However, we did also observe several of the larger degradation products as shown in Fig. 5C (and Fig. S11). The lack of the smaller degradation products as initially observed in the bead system (Fig. 3A) could be explained by the reduced incubation time of 24 hours compared to seven days in the beads. We also observed small amounts of the two features (*m/z* 901.5862 and *m/z* 814.5541) in the culture with *Chryseobacterium* sp*.* D764. This could indicate that *Chryseobacterium* sp*.* D764 also secretes enzymes that can break down **2**, although to a much lesser extent than *S. indicatrix* D763. Thus, the fate of orfamide A (**1**) in our experimental setup was affected by a sequential, multi-species interaction involving the initial hydrolysis by *R. globerulus* D757 and subsequent degradation by primarily *S. indicatrix* D763 (Fig. 5D).

## Discussion

In this study, we utilized a four-membered bacterial SynCom cultivated in an artificial hydrogel bead system to evaluate the contribution of the cyclic lipopeptide, orfamide A, from a *P. protegens* strain on the ability of the *Pseudomonas* to invade and establish within the simplified microcosm. Although the genotypic complexity of our four-membered SynCom is far from a representation of the natural soil microbial diversity [33, 34], the simplified system applied in this study allowed for the systematic analysis of community-level interactions affecting the secondary metabolome of *P. protegens* DTU9.1. We demonstrated that *P. protegens* readily invaded and altered the community composition. Unexpectedly, the knockout mutant unable to produce orfamide A invaded the community as efficiently as the wildtype did and caused similar perturbations in the composition of the synthetic community (Fig. 2), despite the observation that one SynCom member was sensitive to orfamide A (Table 1). This was not a result of an inability to produce non-toxic levels of orfamide A, as levels of orfamide A exceeded the MIC value towards at least one SynCom member during axenic cultivation of *P. protegens* DTU9.1 in the hydrogel bead system (Table 2). Similarly, previous studies reported the lack of advert effects of the biocontrol strain *P. protegens* CHA0 on indigenous microorganisms in situ, despite large fractions of subsequently isolated rhizobacteria displaying sensitivity towards antimicrobial metabolites produced by the biocontrol strain in vitro [11, 35]. This could suggest that sensitive bacteria either colonize distinct niches separated from the antibiotic-producing biocontrol strain in situ or that naturally co-occurring microorganisms sustain toxic levels of antimicrobial metabolites via community-level tolerance mechanisms, such as multi-species biofilm formation [36] or enzymatic inactivation of antimicrobial metabolites [37].

Microbial interactions among co-existing microorganisms have been studied extensively over the past decades to identify and understand the diverse means by which these microbes communicate and compete. Foster and Bell reported that interspecies competition among bacteria isolated from the same sample site was the far most dominant type of interaction [38]. This could suggest that bacteria have evolved intricate sensing mechanisms to respond to danger cues secreted by competing organisms [39, 40]. In our study, we found that levels of the antimicrobial metabolites, DAPG and pyoluteorin, produced by the invading *P. protegens* were elevated during cocultivation with the four-membered SynCom (Table 2). This suggests that the biosynthesis of these metabolites is induced in *P. protegens* as a response to cues secreted by one or more competing members of the SynCom, which we have observed previously in the case of pyoluteorin biosynthesis [41]. The concentration of both DAPG and pyoluteorin after seven days of cultivation (Table 2) exceeded the minimal inhibitory concentration towards several SynCom members (Table 1). We further discovered that a mutant of *P. protegens* DTU9.1 deficient in production of DAPG, pyoluteorin, and orfamide A (ΔTriple) was unable to establish and reach cell titers comparable to its wildtype derivative during coculture with the SynCom (Fig. 2A and 2D). However, knockout mutants deficient in either DAPG or pyoluteorin production invaded the SynCom as efficiently as wildtype *P. protegens* (Fig. S5A). Biosynthesis of DAPG and pyoluteorin is intricately regulated in *P. protegens* and is interlinked due to its shared dependency on the precursor, phloroglucinol [42, 43]. Thus, one could imagine a compensatory effect upon generation of individual knockout mutants (DAPG levels would increase as a result of knocking out *pltA*, and vice versa). This was consistent with what we observed during axenic cultivation of the Δ*phlACB* and Δ*pltA* mutants (Fig. S5B), which may explain the absence of any noticeable effect on community composition over time when the mutants were introduced into the SynCom. Our results suggest that *P. protegens* DTU9.1 utilizes its secreted antimicrobial secondary metabolites to establish within the reduced four-membered microbiome. Specifically, we show that DAPG and pyoluteorin are required for establishment, whereas we cannot exclude that orfamide A also play a role in colonization. However, in our hydrogel bead setup orfamide A is inactivated and catabolized by SynCom members, and thus never reaches levels expected to have a significant impact.

Lipopeptides from fluorescent *Pseudomonas* are versatile metabolites known for their antimicrobial properties and involvement in bacterial motility [44, 45]. Particularly, Gram-positive bacteria have been associated with increased susceptibility towards lipopeptides, due to the lack of a protective cell wall [46], thus it is not surprising that some Gram-positive Actinobacteria have evolved resistance mechanisms towards lipopeptides involving enzymatic inactivation [47, 48]. In our study we discovered that the Gram-positive *R. globerulus* D757 inactivated orfamide A by hydrolysis of the thermodynamically sensitive ester bond connecting the macrocyclic ring. According to D’Costa and colleagues, hydrolysis of the ester bond is the most common resistance mechanism by which Actinobacteria enzymatically inactivated the cyclic lipopeptide, daptomycin [48]. Although enzymatic inactivation of orfamide A is likely a resistance mechanism in *R. globerulus* D757, we also demonstrated that hydrolysis caused a significant inhibition of the swarming motility of *P. protegens* DTU9.1. This indicates that the ability of *R. globerulus* D757 to hydrolyze the cyclic lipopeptide prevents swarming-mediated spread of *P. protegens*.

We also observed that biotransformation of orfamide A by *R. globerulus* D757 was a prerequisite for subsequent degradation by *S. indicatrix* D763 (Fig. 5). Two recent studies have similarly demonstrated how biotransformation of *Pseudomonas*-produced cyclic lipopeptides can have dramatic effects on their chemical and ecological properties [30, 49]. The first found that coculture between a *Pseudomonas* and a *Paenibacillus* strain led to the enzymatic modification of syringafactin, thus changing the chemical structure of the lipopeptide resulting in an amoebicidal byproduct [30]. The second study demonstrated that Gram-positive *Mycetocola* strains could disarm the activity of the mushroom pathogen, *Pseudomonas tolaasii*, by hydrolysis of the ester bonds in the two *Pseudomonas*-produced cyclic lipopeptides, tolaasin I and pseudodesmin A, thus preventing pathogenesis [49]. In our study, we identified catabolism of hydrolyzed orfamide A (Fig. 3A and Fig. 5), which suggests that *S. indicatrix* D763 might utilize the hydrolyzed orfamide A as an alternative nutrient source. A prior study has demonstrated the ability of isolated soil bacteria to utilize penicillin as sole carbon source to support growth by enzymatically catabolizing the antibiotic [50]. In our hydrogel bead setup, we did not observe any noticeable growth benefit for *S. indicatrix* D763 resulting from orfamide A catabolism when comparing the two systems (SynCom + WT and SynCom + Δ*ofaA*). This is perhaps not surprising considering the availability of nutrients in the supplemented 0.1x TSB, even after seven days. Further investigation of the impact of orfamide A catabolism will be the subject for future studies.

Previous research showed that *Pseudomonas*-produced cyclic lipopeptides are rapidly degraded when added exogenously to non-sterile soil yet remain stable in sterilized soil, which clearly suggests that unknown members of the indigenous soil microbiome possess the ability to transform and/or degrade metabolites with activities relevant for biocontrol [51]. Our discovery and characterization of community-level inactivation and degradation of orfamide A involving two co-occurring soil bacteria in our SynCom provides a mechanistic explanation for this observation. Although the prevalence of such processes in different soil communities is currently unknown, we suggest that biotransformation processes of cyclic lipopeptides and other biocontrol metabolites [52] may contribute to variations in efficiency in biocontrol applications. Collectively, our results illustrate the usefulness of synthetic communities to systematically investigate how microbial communities respond to antibiotics to enhance their resilience towards microbial invasion, and to identify processes that determines the turnover and “fate” of biocontrol metabolites. Improved knowledge of potential constraints in efficient biocontrol is a prerequisite for development of efficient biocontrol products or for development of measures to counteract the community processes responsible for modification and degradation of biocontrol metabolites.

## Supplementary Material

Supplementary_clean_wrae105
