# Supplementary material for: Resistance towards and biotransformation of a Pseudomonas-produced secondary metabolite during community invasion
Source: ISME J. 2024 Jun 14;18(1):wrae105. doi: 10.1093/ismejo/wrae105 (PMC11203913; doi:10.1093/ismejo/wrae105)
Supplement: Supplementary_clean_wrae105 [file supplementary_clean_wrae105.pdf]

Supplementary file

Resistance towards and biotransformation of a *Pseudomonas*-produced secondary metabolite during community invasion

Morten L. Hansen, Zsófia Dénes, Scott A. Jarmusch, Mario Wibowo, Carlos N. Lozano-Andrade, Ákos T. Kovács, Mikael L. Strube, Aaron J. C. Andersen and Lars Jelsbak[#].

Department of Biotechnology and Biomedicine, Technical University of Denmark, Søltofts Plads bldg. 221, DK-2800 Kgs Lyngby, Denmark

Supplementary methods

Measurement of growth rates

To calculate growth rates of each SynCom member, as well as the two variants of *P. protegens* DTU9.1 (WT and Δ*ofaA*) all bacteria were cultured in four biological replicates in LB broth O/N. Cells were washed twice in 0.9% NaCl. A clear 96-well flat-bottom microplate (Greiner Bio-One) was prepared with 200 µl 0.1x Tryptic Soy Broth (TSB, Sigma) per well inoculated with bacteria to an initial optical density at 600 nm (OD_{600}) of 0.01. The microplate was covered with semi-permeable membrane (Breathe-Easy, Merck) and incubated in a Cytation5 microplate reader (BioTek Instruments) at 22°C and continuous shake. Measurements of optical density at OD_{600} were taken every 10 minutes for 24 hours. Growth rates were estimated with a linear regression during exponential growth and compared statistically using a one-way ANOVA with Tukey's multiple comparison test in GraphPad Prism 10.

DNA extraction from hydrogel bead systems and 16S rRNA gene amplicon sequencing

DNA from the hydrogel bead systems run in three biological replicates were extracted after 1, 4 and 7 days with a DNAeasy PowerSoil kit (Qiagen) following the manufacturer's instructions. The V3-V4 region of the 16S rRNA gene was amplified in a 25 µl PCR (5.9 µl Sigma Water, 12.5 µl 2x TEMPase, 0.8 µl 10 µM barcoded 16S-341F, 0.8 µl 10 µM barcoded 16S-805R [1], and 5 µl template DNA). The program for the PCR amplification was as follows: (i) 15 min at 95°C, (ii) 30 cycles of 30 sec at 95°C, 30 sec at 60°C, and 30 sec at 72°C, and (iii) 5 min at 72°C. Amplicons were purified with the Agencourt AMPure XP kit (Beckman Coulter) and eluted in 10 mM Tris-HCl (pH = 8.5). Following purification, the amplicons were pooled in equimolar concentrations and shipped to Novogene (Cambridge, United Kingdom) for 250PE sequencing on an NovaSeq 6000 (Illumina) platform with 3 Gb raw data per sample. The raw reads were demultiplexed using cutadapt v3.7 [2]. We used *DADA2* 1.16 [3] in *R* 4.0.2 to denoise, join reads and perform downstream analyses with the scripts generated by Henriksen et al. [4]. Relative read abundance was normalized to the number of 16S rRNA genes per genome.

Using flow cytometry to estimate population dynamics in swarming colonies

Prior to setting up the swarming assay the two variants of *P. protegens* DTU9.1 (WT and Δ*ofaA*) were chromosomally tagged with a constitutively expressed *gfp* gene using pBG42, which is a Tn7-based plasmid from Zobel et al. [5]. This would allow us to distinguish between non-fluorescent *R. globerulus* D757 and green fluorescent *P. protegens* DTU9.1. The swarming assay was conducted according to the main text (see Methods). After 48 hours of incubation on 0.1x TSA plates with 0.6% agar, pictures were taken for analysis and microbial biomass was collected by flushing the surface of the agar plates with 1 ml 1x PBS buffer. The cell suspensions were diluted appropriately before analysis on a MACSquant flow cytometer (Miltenyi Biotec).

Supplementary references

1. Klindworth A, Pruesse E, Schweer T, Peplies J, Quast C, Horn M, et al. Evaluation of general 16S ribosomal RNA gene PCR primers for classical and next-generation sequencing-based diversity studies. *Nucleic Acids Res* 2013; **41**: e1.

2. Martin M. Cutadapt removes adapter sequences from high-throughput sequencing reads. *EMBnet J* 2011; **17**: 10–12.

3. Callahan BJ, McMurdie PJ, Rosen MJ, Han AW, Johnson AJA, Holmes SP. DADA2: High-resolution sample inference from Illumina amplicon data. *Nat Methods* 2016; **13**: 581–583.

4. Suhr NN, Henriksen E, Schostag MD, Rosen Balder S, Bech PK, Strube ML, et al. The ability of *Phaeobacter inhibens* to produce tropodithietic acid influences the community dynamics of a microalgal microbiome. *ISME Communications* 2022; **2**: 109.

5. Zobel S, Benedetti I, Eisenbach L, De Lorenzo V, Wierckx N, Blank LM. Tn7-Based Device for Calibrated Heterologous Gene Expression in *Pseudomonas putida*. *ACS Synth Biol* 2015; **4**: 1341–1351.

Supplementary Table 1

Name	Sequence^{a,b,c}	Note
***phlACB* deletion**		
Up_F_{phlA}	5’- atcccgtctagaCAGAGATTTCGCAGTAAAAAG	Amplification of homology region upstream of *phlA*
Up-R_{phlA}	5’- **catcgacgatttccgaagcgatcac**TTTCCTCTTGATTCCATTCTTTTC	Amplification of homology region upstream of *phlA*
Down_F_{phlB}	5’- GTGATCGCTTCGGAAATCG	Amplification of homology region downstream of *phlB*
Down_R_{phlB}	5’- atccgggagctcACAACGAGGAGAATTCCAG	Amplification of homology region downstream of *phlB*
phlACB-del_fw	5’- GTGCGAGTTCAATCATCTGG	Verification of *phlACB* deletion
phlACB-del_rev	5’- CTCTCGTAGTTGAGCCGTTC	Verification of *phlACB* deletion
***pltA* deletion**		
Up_F_{pltA}	5’-atcccgtctagaAGCGCCTTCATTCCTAAATC	Amplification of homology region upstream of *pltA*
Up-R_{pltA}	5’- **gatgccaaagtaatgcgcatcgaag**TGCCCACTCCCTGTTAGGC	Amplification of homology region upstream of *pltA*
Down_F_{pltA}	5’- CTTCGATGCGCATTACTTTG	Amplification of homology region downstream of *pltA*
Down_R_{pltA}	5’- atccgggagctcCTGTCCAGCGAAGAGAGTT	Amplification of homology region downstream of *pltA*
pltA-del_fw	5’- GTTCTTTGCATGTTCGAGAAAGAGCAG	Verification of *pltA* deletion
pltA-del_rev	5’- GGGAACGCTTCCAGTCCACC	Verification of *pltA* deletion
***ofaA* deletion**		
Up_F_{ofaA}	5’- atcccgtctagaCTGCGACAGGCTCTTGAGAAAC	Amplification of homology region upstream of *ofaA*
Up-R_{ofaA}	5’- **ccggcgcggggaaaagcacttgcagcggcc**TTCATGCGCGCCCCC	Amplification of homology region upstream of *ofaA*
Down_F_{ofaA}	5’- GGCCGCTGCAAGTGCTTTTC	Amplification of homology region downstream of *ofaA*
Down_R_{ofaA}	5’- atccgggagctcCCAGATGGTTGCGAAACGGTAC	Amplification of homology region downstream of *ofaA*
ofaA-del_fw	5’- ACAGGCGCTCATCGAACTCC	Verification of *ofaA* deletion
ofaA-del_rev	5’- TCAGCCGGGCCTCTTCGATC	Verification of *ofaA* deletion
**16S rRNA amplification**		
16S-341F	5’- CCTACGGGNGGCWGCAG	Amplification of the V3-V4 region of the 16S rRNA gene
16S-805R	5’- GACTACHVGGGTATCTAATCC	Amplification of the V3-V4 region of the 16S rRNA gene

a. CAPITAL letters represent the priming part

b. Underlined characters represent restriction sites attached as primer overhang

c. **Bold** characters represent the first 25 nucleotides downstream of the region targeted for deletion to achieve successful overlap-extension PCR

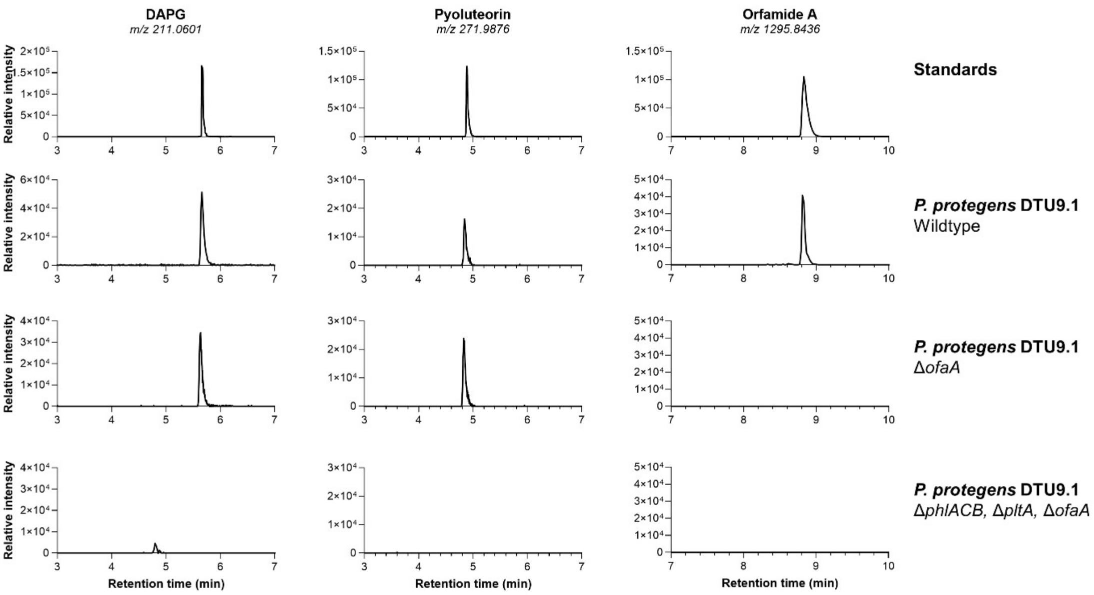

Figure S1 | **Orfamide A detection in hydrogel beads during axenic cultivation of *P. protegens* DTU9.1 variants.** Extracted ion chromatograms (EIC) for DAPG (*m/z* 211.0601 ± 5 ppm), pyoluteorin (*m/z* 271.9876 ± 5 ppm), and orfamide A (*m/z* 1295.8436 ± 5 ppm) detected in hydrogel beads after 7 days of cultivation with *P. protegens* DTU9.1 variants grown axenically.

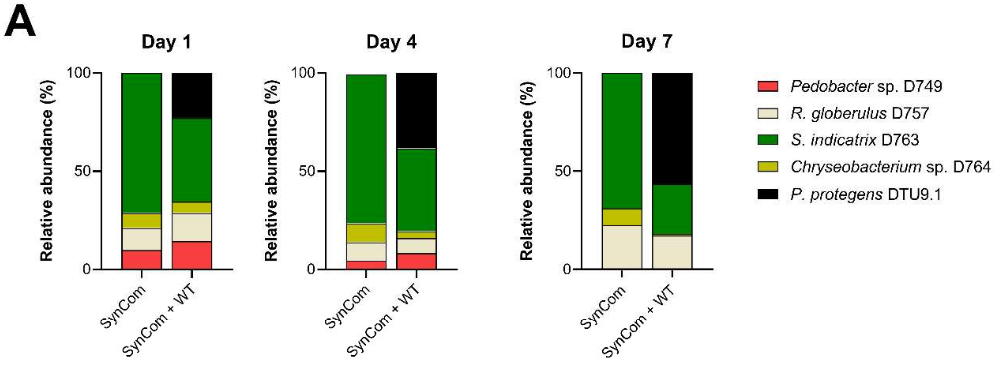

B

Relative abundances in percentages (Mean ± SD)	Day 1		Day 4		Day 7	
	SynCom	SynCom + WT	SynCom	SynCom + WT	SynCom	SynCom + WT
Pedobacter sp. D749	9.95 ± 3.18	14.44 ± 0.74	4.77 ± 1.50	8.40 ± 0.83	0.04 ± 0.02	0.38 ± 0.19
R. globerulus D757	11.12 ± 1.77	14.29 ± 0.48	9.50 ± 1.29	7.60 ± 0.39	22.53 ± 4.16	16.79 ± 3.49
S. indicatrix D763	71.19 ± 5.36	42.62 ± 2.32	76.02 ± 8.67	42.17 ± 2.44	68.96 ± 8.94	25.26 ± 2.77
Chryseobacterium sp. D764	7.74 ± 1.64	5.80 ± 1.28	9.71 ± 6.06	3.57 ± 0.91	8.47 ± 4.77	0.94 ± 0.19
P. protegens DTU9.1		22.84 ± 2.49		38.26 ± 2.66		56.64 ± 3.91

Figure S2 | **Amplicon sequencing of the 16S rRNA gene of the SynCom with and without *P. protegens* DTU9.1 invasion. A)** Relative abundances of each bacterial species after 1, 4 and 7 days of cultivation within a hydrogel bead system. Number of reads were normalized to the presence of 16S rRNA genes per genome. Stacked bars represent the mean of three biological replicates. **B)** Relative abundances in percentages plotted in **A)**. Values represent mean and standard deviation from the three biological replicates.

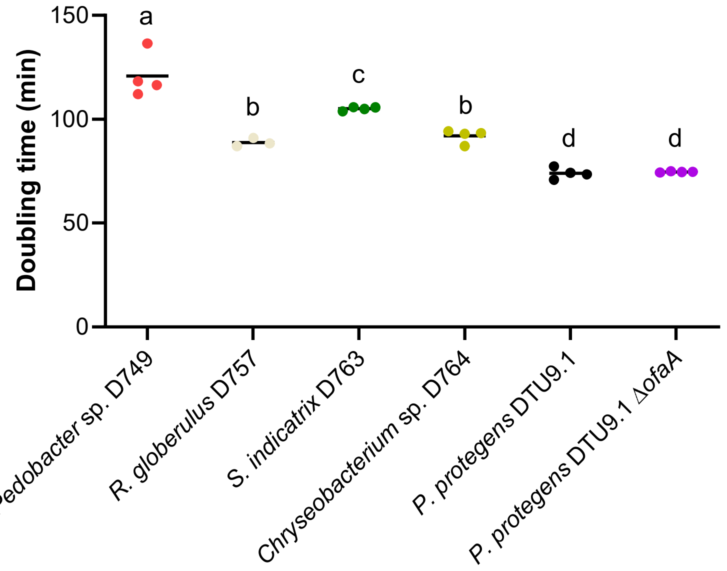

Figure S3 | **Growth assay of SynCom members and *P. protegens* DTU9.1 WT and Δ*ofaA*.** Doubling times of *Pedobacter* sp. D749, *R. globerulus* sp. D757, *S. indicatrix* D763, and *Chryseobacterium* sp. D764., as well as *P. protegens* DTU9.1 WT and Δ*ofaA* in 0.1x TSB liquid broth. Data was derived from 4 biological replicates. Letters above indicate treatments significantly different from one another, as determined by a one way ANOVA with Tukey's multiple comparison test ($P < 0.05$).

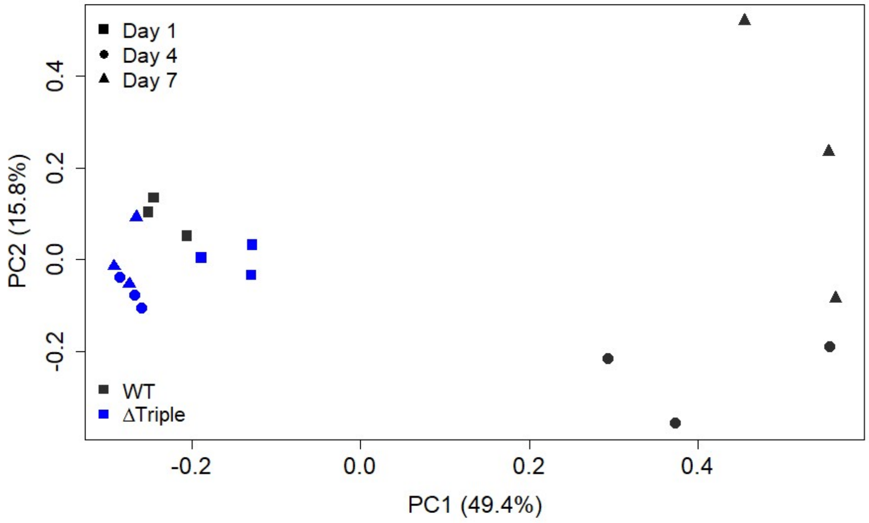

Figure S4 | **PCoA confirms that WT and ΔTriple cause significantly different alterations to overall community abundance.** A Principal Coordinate Analysis (PCoA) was performed on CFU data including all bacterial species of the systems inoculated with *P. protegens* DTU9.1 WT and ΔTriple to compare the effects of sampling time (symbols) and the two variants of *P. protegens* DTU9.1 (color) on the bacterial composition in a soil-like environment. An overall PERMANOVA using sampling time, genotypic variant of invading *P. protegens* DTU9.1, and their interaction as fixed effects further confirmed the significance of genotypic variant on the community composition ($P = 9.99 \cdot 10^{-5}$, $R^2 = 0.31$).

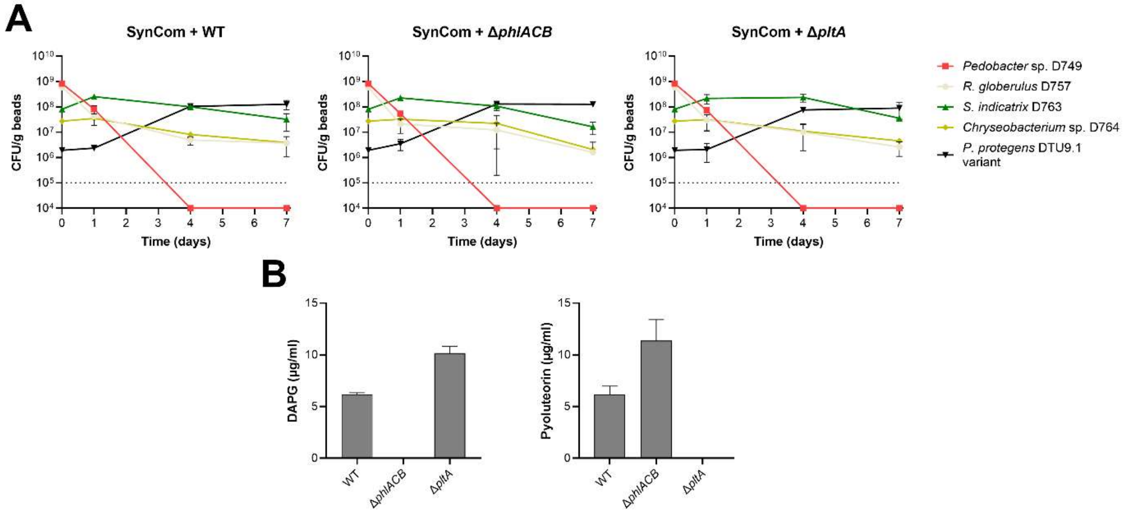

Figure S5 | **Mutants of *P. protegens* DTU9.1 deficient in DAPG and pyoluteorin production invade the SynCom as efficiently as the wildtype.** A) Abundance was determined as colony forming units in the hydrogel bead system of each SynCom member and the introduced *P. protegens* DTU9.1 variant; WT, Δ*phlACB*, and Δ*pltA* after 1, 4, and 7 days. The dotted line represents the limit of detection of colony forming units at 10^5 CFU/ml. Data was derived from three biological replicates. B) Concentration of DAPG and pyoluteorin from *P. protegens* DTU9.1 after 7 days of axenic growth in the hydrogel bead system. Data derived from two replicates.

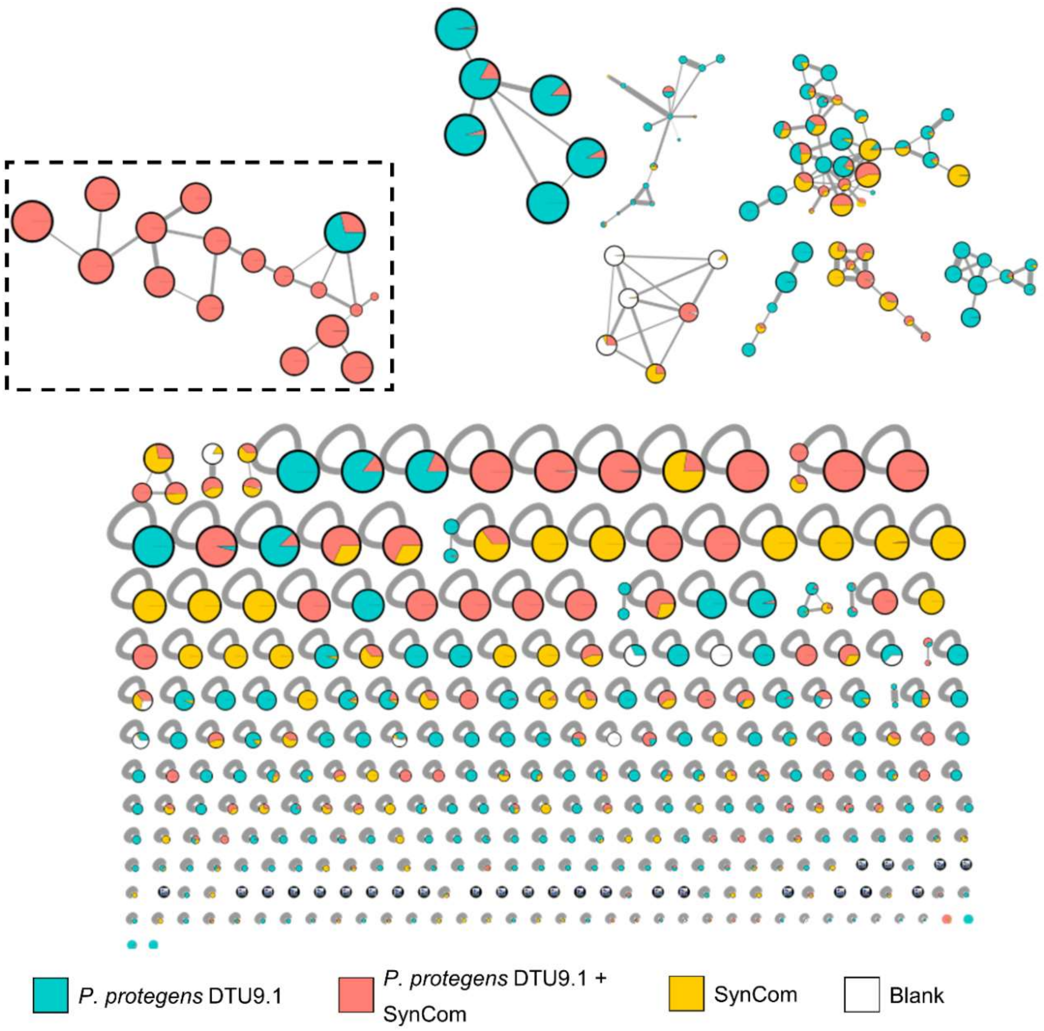

Figure S6 | **Global Natural Products Social network analysis of secondary metabolites extracted from bead systems.** Complete GNPS network on the MS data of the extracts from bead systems grown with monoculture of *P. protegens* DTU9.1 (Cyan), coculture between *P. protegens* DTU9.1 and SynCom (Red), or SynCom by itself (Yellow). White-colored nodes represent instrument blank noise. Nodes represent individual metabolites and the internal pie diagrams display relative abundance. The size of the nodes is scaled to metabolite mass. Edges between nodes represent the similarities between metabolites based on cosine score. The highlighted molecular family contains orfamide A and its respective degradation products identified during coculture.

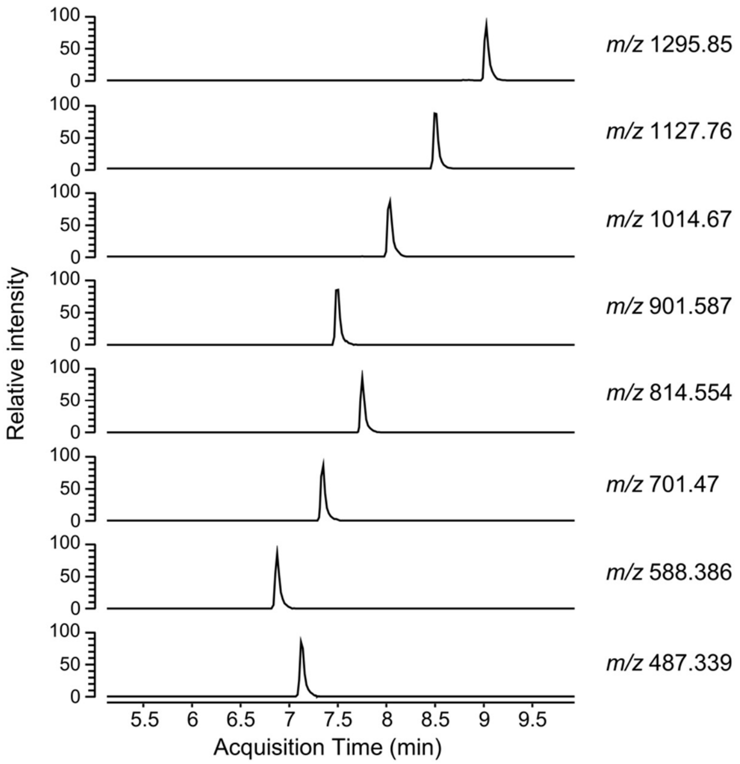

Figure S7 | **Extracted ion chromatograms confirmed the presence of degradation products.** Extracted ion chromatograms (EIC) of orfamide A and the observed degradation products after 7 days of cocultivation between *P. protegens* DTU9.1 and the SynCom in the hydrogel bead system. Each degradation product has a different retention time, thus denying the possibility of them being products of in-source fragmentation.

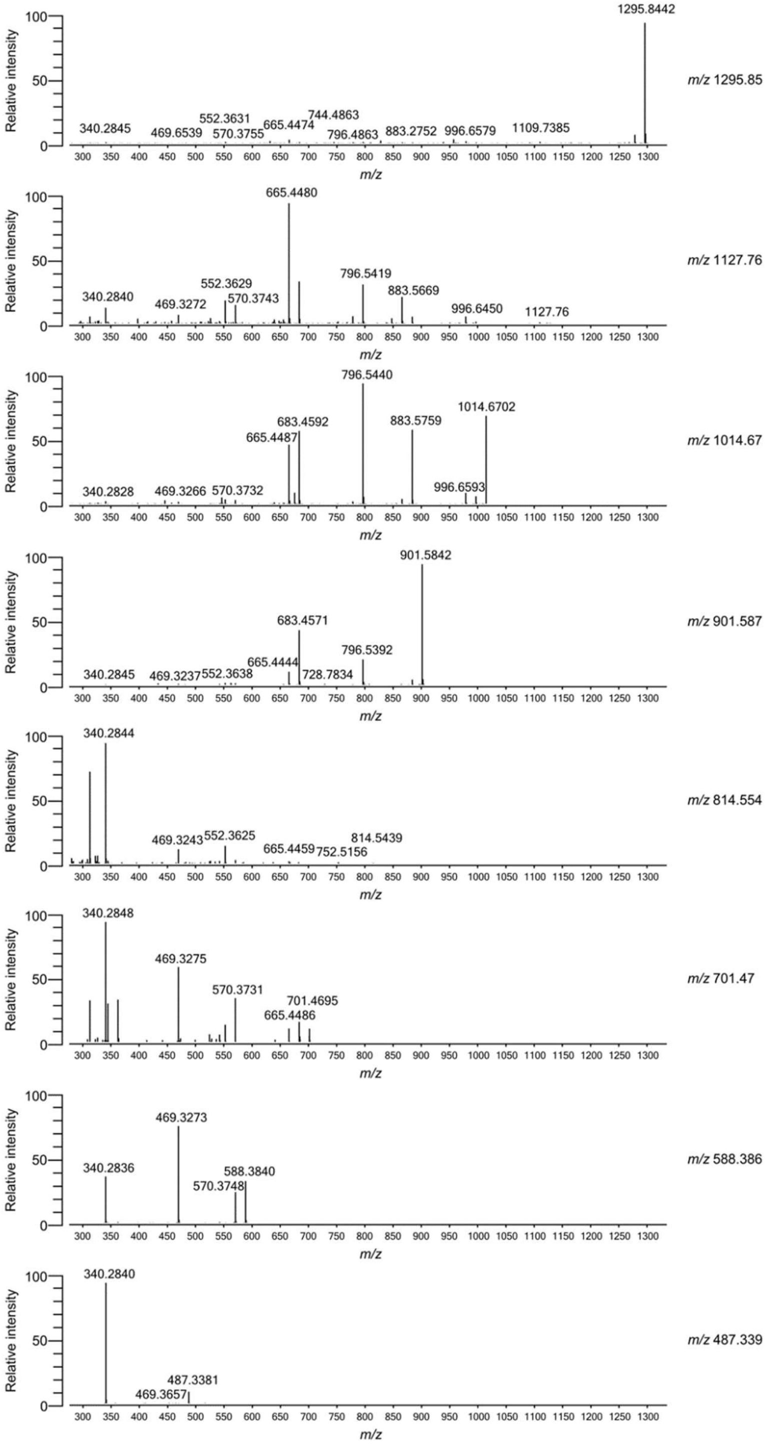

Figure S8 | **Tandem mass spectrometry revealed the relatedness of degradation products to orfamide A.** Observed fragmentation patterns of orfamide A (*m/z* 1295.85) and the degradation products. Similarities between the patterns confirmed the relatedness of each degradation product to orfamide A, representing the loss of amino acids from the C-terminal end of the linearized lipopeptide.

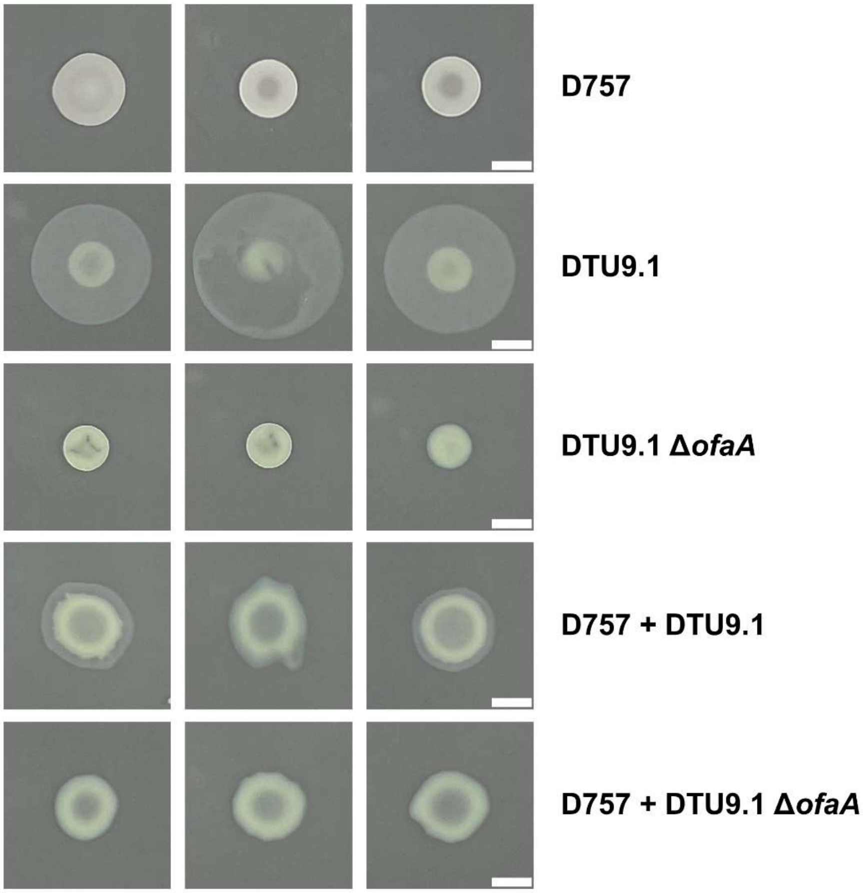

Figure S9 | **Swarming pictures with *P. protegens* DTU9.1 and *R. globerulus* D757.** Swarming areas were determined with ImageJ. Scale bar represents 5 mm. Pictures were taken after 48 hours post inoculation on 0.1x TSA with 0.6% agar. Data is from three biological replicates.

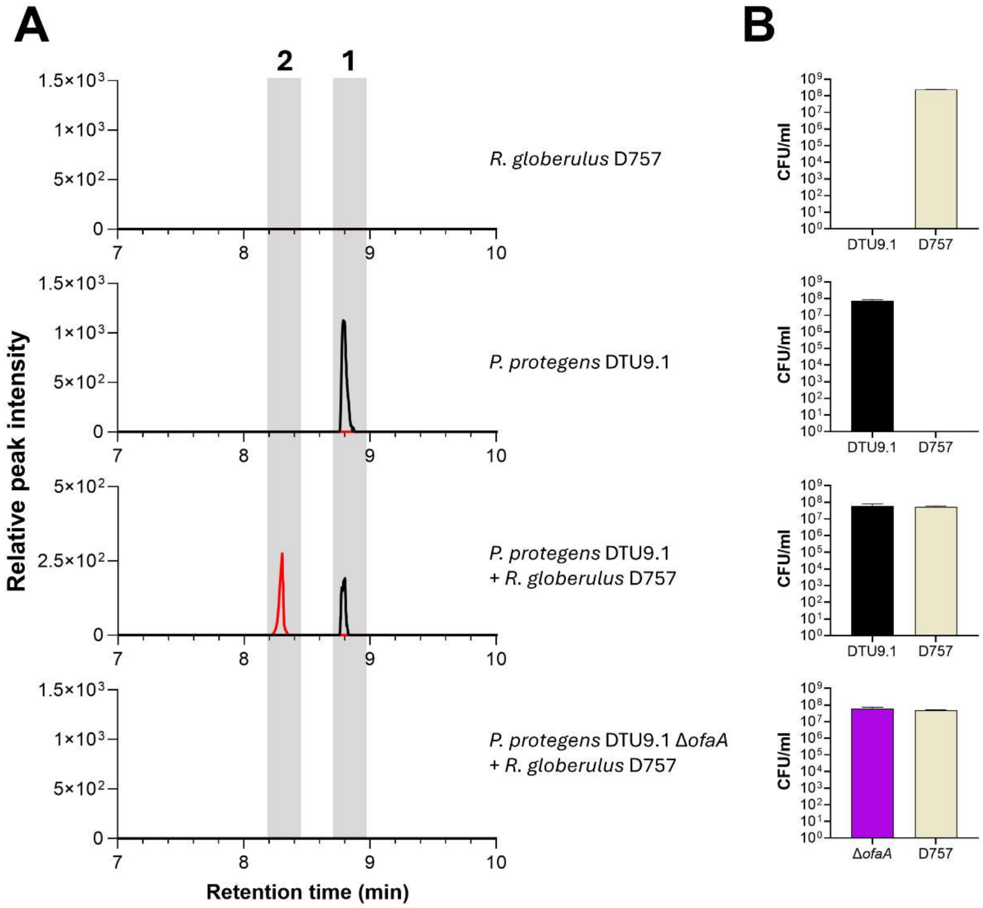

Figure S10 | **Metabolite and flow cytometry analysis of the swarming assay with *P. protegens* DTU9.1 and *R. globerulus* D757.** A) Extracted ion chromatograms (EIC) for 1 (black, *m/z* 1295.8436 ± 5 ppm) and 2 (red, *m/z* 1313.8542 ± 5 ppm) detected in agar plugs from co-cultures of *P. protegens* DTU9.1 and *R. globerulus* D757 on 0.1x TSA with 0.6% agar. B) Abundance of green fluorescent *P. protegens* DTU9.1 and non-fluorescent *R. globerulus* D757 as determined by flow cytometry. Bars represent mean and standard deviation from three biological replicates.

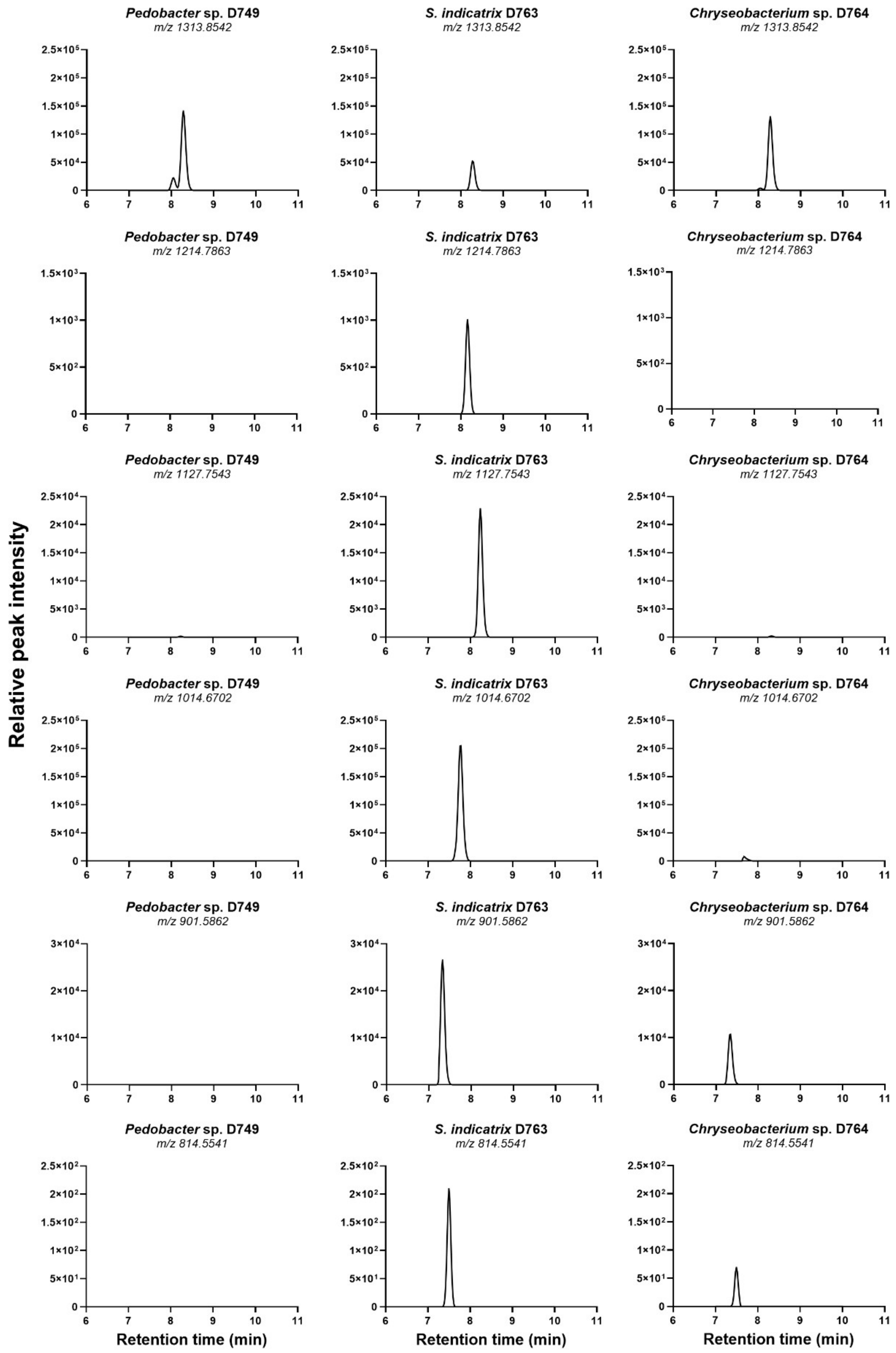

Figure S11 | **Extracted ion chromatograms confirmed *S. indicatrix* D763 as primary degrader of hydrolyzed orfamide A.** Extracted ion chromatograms (EIC) of hydrolyzed orfamide A and the observed degradation products after 24 hours of cultivation *Pedobacter sp.* D749, *S. indicatrix* D763, and *Chryseobacterium sp.* D764 in 0.1x TSB supplemented with pre-hydrolyzed orfamide A. Peaks represent average peak intensities of two biological replicates.
